# The emerging role of cancer nanotechnology in the panorama of sarcoma

**DOI:** 10.3389/fbioe.2022.953555

**Published:** 2022-10-17

**Authors:** Laura Mercatali, Silvia Vanni, Giacomo Miserocchi, Chiara Liverani, Chiara Spadazzi, Claudia Cocchi, Chiara Calabrese, Lorena Gurrieri, Valentina Fausti, Nada Riva, Damiano Genovese, Enrico Lucarelli, Maria Letizia Focarete, Toni Ibrahim, Luana Calabrò, Alessandro De Vita

**Affiliations:** ^1^ Osteoncology Unit, Bioscience Laboratory, IRCCS Istituto Romagnolo per lo Studio dei Tumori (IRST) “Dino Amadori”, Meldola, Italy; ^2^ Clinical and Experimental Oncology, Immunotherapy, Rare Cancers and Biological Resource Center, IRCCS Istituto Romagnolo per lo Studio dei Tumori (IRST) “Dino Amadori”, Meldola, Italy; ^3^ Department of Chemistry “Giacomo Ciamician”, University of Bologna, Bologna, Italy; ^4^ Osteoncologia, Sarcomi dell’osso e dei tessuti molli, e Terapie Innovative, IRCCS Istituto Ortopedico Rizzoli, Bologna, Italy

**Keywords:** sarcoma, nanotechnology, lipid-based nanocarriers, polymeric nanoparticles, smart materials

## Abstract

In the field of nanomedicine a multitude of nanovectors have been developed for cancer application. In this regard, a less exploited target is represented by connective tissue. Sarcoma lesions encompass a wide range of rare entities of mesenchymal origin affecting connective tissues. The extraordinary diversity and rarity of these mesenchymal tumors is reflected in their classification, grading and management which are still challenging. Although they include more than 70 histologic subtypes, the first line-treatment for advanced and metastatic sarcoma has remained unchanged in the last fifty years, excluding specific histotypes in which targeted therapy has emerged. The role of chemotherapy has not been completely elucidated and the outcomes are still very limited. At the beginning of the century, nano-sized particles clinically approved for other solid lesions were tested in these neoplasms but the results were anecdotal and the clinical benefit was not substantial. Recently, a new nanosystem formulation NBTXR3 for the treatment of sarcoma has landed in a phase 2-3 trial. The preliminary results are encouraging and could open new avenues for research in nanotechnology. This review provides an update on the recent advancements in the field of nanomedicine for sarcoma. In this regard, preclinical evidence especially focusing on the development of smart materials and drug delivery systems will be summarized. Moreover, the sarcoma patient management exploiting nanotechnology products will be summed up. Finally, an overlook on future perspectives will be provided.

## Graphical Abstract

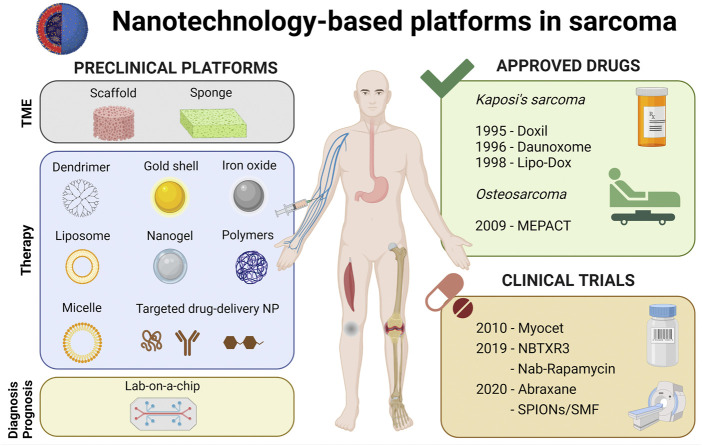

## 1 Introduction

The term sarcoma refers to a wide range of solid lesions accounting for 1% of all adult cancers. These rare neoplasms have been identified on the basis of the affected tissues including cartilage, muscle, connective, adipose, synovial tissue, nerves and bones (Fletcher et al., 2020). Considering the localized disease, the current standard of care is represented by radical surgery with the primary goal of clear margins combined with (neo)adjuvant treatments in selected cases. Although clear margins remain the primary goal of surgical resection, specific anatomy sites of some of these lesions make it difficult to be achieved, especially in the retroperitoneum, thus adjuvant radiotherapy may be an option. In the metastatic setting the gold standard is chemotherapy but the outcomes are still very limited. In this regard, anthracyclines-based regimen represents the first line standard for the treatment of advanced and metastatic soft tissue sarcoma with only 16%–27% response rate when used as single agents (Gronchi et al., 2021). Considering osteosarcoma, the most diffused bone sarcoma, doxorubicin/cisplatin/high-dose methotrexate (MAP) regimen or regimens combining doxorubicin, cisplatin and potentially ifosfamide are the most frequently used as front-line chemotherapy in children and young adult patient, but the outcomes are poor with a 5-year post-relapse survival rate of <20% (Strauss et al., 2021). Therefore, there is a pressing need to develop new therapeutic strategies to improve sarcoma patient outcomes. In this regard, nanotechnology has attracted physicians’ interest offering promising strategies to deliver anticancer therapeutics to tumors. From the magic bullet theory firstly formulated by Paul Ehrlich at the beginning of the 20th century, a long way has come and nanotechnology has landed in the oncology landscape. The use of nano-sized drug delivery systems (DDS) actually is exploited as one strategy to improve the pharmacokinetics properties and tumor specificity together with the circumvention of multiple drug resistance mechanisms including reduced drug uptake (Gavas et al., 2021). On this way, the PEGylated nano-liposomal doxorubicin Doxil^®^ (Caelyx^®^ in Europe) was the first FDA-approved nano-drug in 1995 for the treatment of metastatic breast cancer and metastatic ovarian cancer (Barenholz, 2012). After that, several formulations have been authorized for clinical use. Liposomal daunorubicin DaunoXome^®^ was approved by FDA in 1996 for the treatment of Kaposi’s sarcoma (Mukwaya et al., 1998). Non-pegylated liposomal doxorubicin (Myocet) was approved in 2000 for the treatment of metastatic breast cancer (Mross et al., 2004). In 2005 was approved Abraxane^®^, an albumin-bound paclitaxel, which is used for advanced or metastatic breast cancer (Miele et al., 2009). Genexol PM^®^, a polymeric nanoparticle (NP) micelle formulation of paclitaxel, has been approved in South Korea to treat metastatic breast cancer and is under investigation in a phase II clinical study in the United States to treat pancreatic cancer (Kim et al., 2007). The liposomal irinotecan Onivyde^®^ has been approved by FDA in 2015 for the treatment of pancreatic cancer (Passero et al., 2016). The DDS described above are just a summary of some of the most relevant nanoparticles for cancer therapy. Current research programs and challenges in nanomedicine are focusing efforts on the validation of targeted nano-sized particles appropriately engineered with monoclonal antibody (Ab) or peptide to specifically target tumor cells offering significant advantages in improving cancer therapeutic efficacy and simultaneously reducing drug toxicity. In the latter case, NPs act as carriers of biologically active molecules, and their role is to deliver conventional or innovative therapeutic agents to the site of the disease. In other approaches, the composition and the intrinsic characteristics of *ad hoc* engineered NPs can be exploited in combination with external stimuli to exert a therapeutic effect. Furthermore, on the side of the improvement of therapeutic strategies, the role of nanomedicine is also emerging in diagnostic and prognostic tools together with smart materials for surgical approach. The latter includes the use of tissue engineering technologies combining autologous or allogeneic grafts and substitutes with mesenchymal stem cells, gene therapy and mechanical stability devices (De Vita et al., 2022a). Finally, great efforts have been made for deepening the study of the natural history of these lesions through nanotechnology-based products including tridimensional support for cell culturing and lab on a chip.

### 1.1 Sarcoma biology

The term sarcoma encompasses a wide range of rare entities of mesenchymal origin arising from soft tissue or bone with an incidence estimated in 1% of all adult cancers. These lesions exhibit an extraordinary diversity in terms of morphological features with more than 100 histotypes, genomic profile ranging from simple to complex karyotypes and clinical behavior varying from indolent to aggressive diseases (Fletcher et al., 2020). In this regard these neoplasms are classified on the basis of the involved connective tissue including among the many: blood vessels (angiosarcoma), fat (liposarcoma), smooth muscle (leiomyosarcoma), bone (osteosarcoma, Ewing’s sarcoma and chondrosarcoma), skeletal muscle (rhabdomyosarcoma), skin (Kaposi’s sarcoma), nerves (neurofibrosarcoma), connective tissue (fibrosarcoma, undifferentiated pleomorphic sarcoma, myxofibrosarcoma), synovial tissue (synovial sarcoma), digestive system (gastrointestinal stromal tumor) (Bongiovanni et al., 2014; Bongiovanni et al., 2015; De Vita et al., 2016; Recine et al., 2017; Fletcher et al., 2020; Vanni et al., 2022). Moreover the majority of sarcomas are grouped into three different grades on the basis of histological features and molecular classifications. This grading system includes the presence of genomic aberrations such as translocations, copy number alterations, losses, amplifications, mutations and the findings from gene expression profiling such as the complexity index or single driver genomic abnormality (Oda et al., 2017; Dufresne et al., 2018; Racanelli et al., 2020). From a biological point of view sarcoma occurrence is the result of a complex process which includes the emergence of a driver oncogenic event followed by secondary oncogenic and epigenetic activation processes and aberrations, together with a permissive tumor microenvironment (Grünewald et al., 2020). This extraordinary diversity in terms of morphological and molecular features is reflected also in the clinical behavior displaying a wide range of different manifestations from low grade to high grade lesions which are characterized by an increased risk of developing distant metastasis (Fletcher et al., 2020).

### 1.2 Current sarcoma therapy

Current strategies include multimodal treatment concepts combining surgery, which represents the mainstay for localized disease, with neo (adjuvant) chemo-radiotherapy in selected cases. The aim of radical resection is to obtain margins free from tumor infiltration (R0 surgery). For the metastatic disease the gold standard is represented by chemotherapy but the outcomes are still very poor and its role is debated. In this regard the emerging role of genomic and transcriptomic profiling has led to a more comprehensive overview of sarcoma biology leading to the development of targeted therapeutics and immunotherapy.

Since the end of the 70’s, the first-line treatment in sarcoma patients with metastatic disease is represented by anthracycline-based regimens (Benjamin et al., 1975; Borden et al., 1987; Judson et al., 2014), excluding some specific histotypes for which specific treatments have been established (i.e., Imatinib in Dermatofibrosarcoma Protuberans) (Rutkowski et al., 2017). An international consensus on the second-line treatment has not been yet established and comprises different chemotherapy including: trabectedin (Kawai et al., 2015; Demetri et al., 2016), pazopanib (van der Graaf et al., 2012), eribulin (Schöffski et al., 2016), gemcitabine-based regimens (Ferraresi et al., 2008; García-Del-Muro et al., 2011; Pautier et al., 2012), high-dose ifosfamide (van Oosterom et al., 2002).

Moreover, targeted therapy currently under clinical investigation or emerging in clinical practice involves the use of small molecules or monoclonal antibodies directed against one or more biomarkers. In this regard the following are some of the most investigated: apatinib (VEGFR), dasatinib (Src, KIT, EPHA2, PDGFR), imatinib (PDGFR, KIT), larotrectenib (NTRK), nilotinib (BCR-ABL, DOR, KIT, PDGFR, M- CSFR), pazopanib (VEGFR, PDGFR, KIT), sorafenib (RET, VEGFR), sunitinib (FLT3, PDGFR, VEGFR, M-CSFR), crizotinib (ALK and/or ROS1), Cediranib (VEGFR) R1507 (IGF1R) bevacizumab (VEGF), trastuzumab (HER2/neu), ridaforolimus (mTOR), STAT3 inhibitors, tocilizumab (IL-6 receptor), MG7112 (MDM2), Tazemetostat (EZH2), Denosumab (RANKL) (Dufresne et al., 2018; Grünewald et al., 2020).

Furthermore, the current experience with immunotherapy using anti-programmed cell death protein 1 (PD-1) antibodies as single agents has generally led to disappointing results in both selected or mixed sarcoma histologies (Burgess and Tawbi, 2015; Ben-Ami et al., 2017; Tawbi et al., 2017; Toulmonde et al., 2018). Otherwise combination treatment of nivolumab and ipilimumab targeting both PD-1 and CTLA-4 has led to an increase in response rate and PFS (D'Angelo et al., 2018). Taking in consideration the above, to date evidences underline the effectiveness of immune-checkpoint inhibition in specific immune and/or molecularly defined subgroups of sarcomas leading to the need of a more extensive characterization of sarcoma immune landscape microenvironment aimed at the design of clinical trials (Tazzari et al., 2021).

## 2 Nanotechnology-based platforms in sarcoma translational research

In this section, preclinical evidence on the emerging role of nanotechnology for the study of sarcoma lesions will be reviewed. Although sarcoma represents a wide range of rare entities, several translational studies have been carried out on these lesions. As a consequence, it is important to distinguish between the studies which focused on the use of smart materials for the study of sarcoma pathophysiology or for the treatment of localized sarcoma, and studies which focused on the development of treatment for advanced and metastatic sarcoma.

### 2.1 Drug delivery systems

Considering the development of new therapeutic strategies for the treatment of advanced and metastatic sarcoma, several DDS have been studied. The interest in their use resides, as previously reported, in their ability in improving therapeutic efficacy and reducing toxicity of chemotherapeutics. This is extremely evident in sarcoma tumors in which the role of chemotherapy has not been completely elucidated and the first-line treatment has remained unchanged, excluding some specific entities, from the 70s to nowadays. Thus, the management of cardiotoxicity associated, for instance, with anthracycline-based regimens or neutropenia represents one of the medical needs that physicians have to deal with. Besides these limitations, several others include the low half-life, solubility and bioavailability of chemotherapy which represent some of the major drawbacks of its use especially in frail patients. In some cases, the use of combinations of chemotherapeutics is crucial to trigger a synergic efficacy of the treatment, as reported by Sabei et al. (2021) who used polymeric nanoparticles to deliver a combinatorial therapy for Ewing’s sarcoma.

For the above scopes, different DDS have been designed which could be mainly divided into passive and active targeting formulations. The first type encompasses all the nanovectors in which specific conditions associated with the tumor, including inflammation, hypoxia and an increase in blood vessels permeability are exploited to concentrate drugs at the tumor site (Rosenblum et al., 2018). Thus, passively targeted DDS relies on unique characteristics of solid tumors including more permeable vasculature and defective lymphatic drainage which allow DDS to preferentially accumulate in the tumor site. This phenomenon, firstly described by Matsumura and Maeda, is called the enhanced permeability and retention (EPR) effect (Matsumura and Maeda, 1986). Starting from this, a number of sarcoma passively targeted DDS have been studied. An example is the study of Chen and colleagues who developed an mPEG-PLA-based nanosystem (Chen et al., 2021) loaded with docetaxel assessing their passive targeting and activity in mice bearing S180 sarcoma tumor. Sasatsu et al. (2008) synthesized a methoxypolyethylene glycol amine-poly (DL-lactic acid) copolymer nanoparticles loaded with pyrene-ended poly (DL-lactic acid) providing evidence of passive sarcoma-180 tumor targeting. Palmityl-D-glucuronide-based liposomes incorporating the antitumor agent dipalmitoylphosphatidylfluorouridine (DPPF) were studied as drug carriers for anticancer agents, in mice bearing subcutaneously implanted osteosarcoma cells in 1994 (Doi et al., 1994).

Other examples of passive DDS have been reported for the treatment of osteosarcoma (OS) to address some critical issues with chemotherapeutic drugs, such as very poor water solubility, elevated systemic toxicity and drug resistance. For example, in preclinical studies, paclitaxel (PTX) linked to albumin nanoparticles (nab-paclitaxel/Abraxane™) has been shown to be more effective than PTX alone (Yang et al., 2012; Wagner et al., 2014). Furthermore, passive targeting could be achieved also *via* external stimulation including the application of light energy or magnetic field.

In this regard, to address multidrug resistance in OS nano DDS which carry photoactivatable drugs alone or in combination with PTX have been developed. Photodynamic therapy (PDT) has been proven to be effective against OS cells *in vitro* and *in vivo* (Zeng et al., 2013; Li et al., 2016; White et al., 2016; Meier et al., 2017). Poly-methyl methacrylate nanoparticles linked to a photosensitizer have proved to be more effective than a photosensitizer alone *in vitro* and *in vivo* (Duchi et al., 2013; Lenna et al., 2020). PDT can also be used as a co-adjuvant therapy for cancer treatment in combination with antineoplastic drugs to enhance treatment outcome. Recently, the generation and efficacy of keratin nanoparticles covalently linked to a photosensitizer have been published. The additive effect of this multimodal approach has been demonstrated in 3D OS models *in vitro* and in an orthotopic model of OS (Martella et al., 2018; Martella et al., 2022). In this case of the application of magnetic field it is referred as magnetic drug targeting. Magnetite-dextran composite nanoparticle-bound mitoxantrone have been developed and their targeting ability through the use of a 0.6 tesla extracorporeal magnets was assessed with promising results in a rhabdomyosarcoma rat model (Krukemeyer et al., 2012). To give a taste of the importance of the EPR mechanism, it is important to consider that all clinically approved cancer nanomedicines belong to the class of passive targeting formulations. Yet, the efficiency of the EPR mechanism is strongly dependent on the type of tumor, on its vascularization, accessibility, and on the specificity and heterogeneity of the tumor microenvironment.

The second group of nano-sized particles gather the ligand mediated targeted nanosystems which represent the active targeting formulations. Unlike passive targeting formulations, these DDS are decorated with antibodies, peptides or molecular moieties to specifically target tumor lesions. Rodríguez-Nogales and colleagues functionalyzed the surface of Squalenoyl-Gemcitabine Nanoparticles with Squalenyl-Hydroxybisphosphonate assessing *in vitro* anticancer activity in human osteosarcoma U2-OS cells and on a patient-derived (531 M) pediatric osteosarcoma cell line (Rodríguez-Nogales et al., 2021). Other active targeting DDS exploit hypoxic conditions surrounding the tumor lesions. This is the case of nanosystems based on oxygen-absorbing perfluorotributylamine (PFA) and tirapazamine synthesized starting from polydopamine (PDA)-coated UiO-66 metal organic framework (MOF) which showed in osteosarcoma murine model (Chen at al., 2021b). Other attractive nanosystems are gold nanoparticles due to their multifunctional applications and biological activities (Hu et al., 2020). In a recent work Naumann and colleagues (Naumann et al., 2018) conjugated gold nanoparticles with topoisomerase I inhibitor SN-38 to assess their efficacy *in vitro* and *in vivo* using Ewing sarcoma cells. Moreover, increasing evidence of the pivotal role of immunomodulation for tumor treatment is emerging. In this regard, the derivatization of chitosan nanoparticles with methylglyoxal has proved to be efficient in terms of antitumor property and elicits macrophage-mediated immunity in Sarcoma-180 tumor bearing mice (Chakrabarti et al., 2014). Other studies focused on the use of peptides as ligands for the active targeting. This is the case of integrin receptor-targeted Lipid-Protamine-siRNA (LPR) nanoparticles study in which Arg-Gly-Asp (RGD) peptide liposomes were used to significantly reduce tumor growth *in vitro* and *in vitro* using alveolar rhabdomyosarcoma cells (Rengaswamy et al., 2016).

Finally, another emerging approach is represented by the use of iron oxide nanoparticles for magnetic resonance and gene therapy. In this regard, a recent work focused on assessment of low-molecular-weight poly (ethylenimine) (PEI)-poly (ethylene glycol) (PEG) nanogels (NGs) delivering transforming growth factor-β1 (TGF-β1) siRNA and ultrasmall iron oxide nanoparticles (Fe_3_O_4_ NPs) for gene therapy and *T*_1_-weighted magnetic resonance (MR) (Peng et al., 2021).

The passive and active approaches are not completely independent; furthermore, they share major common points. First of all, active targeting nanomedicines—to fully exploit their potential—should also pre-accumulate within the solid tumor, i.e., they should also behave as passively targeted formulations. The optimization of this first step is strongly dependent on size, surface chemistry, shape and mechanical properties of the nanosystems (Rosenblum et al., 2018). In addition, both types face the risks related to opsonization, i.e., to the formation of a protein corona which can substantially alter the bio-nano interactions and eventually the fate of the nanomedicine. PEGylation is the most widely accepted method to reduce opsonization, resulting in prolonged circulation time, shielding from immune system and improved bioavailability. Polyethyleneglycol (PEG) lipid, 1,2-distearoyl-sn-glycero-3-phosphoethanolamine-PEG (DDA-PEG) arms have been used for the PEGylation of liposomes overcoming the clearance of the reticuloendothelial system in a M5076 ovarian sarcoma cells mice model (Chen et al., 2017; Sugiyama et al., 2017; Ren et al., 2019; Papini et al., 2020).

### 2.2 Lab-on-a-chip devices

Increasing interest is related to finding novel high-throughput strategies combining microfluidics, bioengineering, nanotechnology and the use of cells or tissue for the development of lab-on-a-chip devices as promising platforms for diagnosis, prognosis and drug screening.

An example of lab-on-a-chip is the work of Charamiec and colleagues (Chramiec et al., 2020) who have developed an integrated microfluidic system for the prediction of anti-tumor drug efficacy and cardiotoxicity for Ewing sarcoma model. A recent work focused on the establishment of a fast and efficient ZnO-nanorods integrated microfluidic chip for the quantification of plasma extracellular vesicles as biomarkers for osteosarcoma patients (Xu et al., 2021). Challenging testing is represented by the possibility to combine the assessment of chemotherapy together with radiotherapy, both representing standard treatments for sarcoma. A recent work reports the design of a polydimethylsiloxane (PDMS) microfluidic device which allowed the assessments of chemotherapeutic and radiotherapeutic cytotoxic and anti-proliferative effects on STS (Patra et al., 2019). The authors have screened the pharmacological and radiotherapeutic profile of two STS cell lines exposing them to different doses of radiotherapy ranging from 0.5 Gy to 8 Gy with doxorubicin at 2 μM and 20 µM concentration observing the cell death with doxorubicin throught apoptosis and through other pathways with RT. Furthermore, another field of application of nanotechnology-based platforms is represented by the manipulation of cells and tissues with the use of devices engineered through nanotopography. In this regard Hulshof reported a nanometer scale feature Nano-TopoChip (Hulshof et al., 2017) able to influence osteosarcoma cell phenotype, morphology, cell spreading and orientation as a high-throughput screening platform. Recently, Hasegawa and colleagues have developed a system for detecting circulating sarcoma cells by On-chip Sort (Hasegawa et al., 2019). In a pilot study the authors have used human fibroblast and sarcoma cell lines as models to design a circulating sarcoma cells separation protocol. Then they applied the protocol to the whole blood from a patient with locally advanced myxofibrosarcoma confirming the validity of the system in separating the circulating sarcoma cells. Furthermore, microchips with printed electrodes have been designed for the detection of viral load in plasma and saliva. Briefly, the authors provided evidence of the ability of the device in detecting and quantifying HIV, Epstein-Barr Virus and Kaposi’s Sarcoma-associated Herpes Virus in small volumes of PBS, plasma and artificial saliva samples. Moreover, they confirmed the validity of the system through the analysis of HIV-infected patient samples (Shafiee et al., 2015). Finally, microfluidic chips loaded with a colorimetric nanoparticle assay have been developed for the detection of Kaposi’s sarcoma associated herpesvirus through a smartphone-based technology (Mancuso et al., 2014).

## 3 Novel three-dimensional culture systems

As reported by Gao et al. (2017) and colleagues new models for sarcoma research are required to improve our understanding of the disease and for developing new therapies. Thus, a widely explored topic is represented by the generation of novel three-dimensional (3D) culture models with the aim of recapitulating the tumor microenvironment for the study of the pathophysiology of sarcoma.

A multitude of 3D scaffolds composed of natural biological biomaterials and/or synthetic polymers have been developed. In this context, improved histocompatibility, mechanical properties, and morphology, including appropriate porosity for cell culture, are some of the features on which 3D culture models are focused. The efforts in scaffold design derived from the need to properly mimic not only the tumor microenvironment (TME) in terms of matrix composition and tridimensionality, but also to imitate the interactions between sarcoma cells and extracellular matrix (ECM), the biomechanical features of growing tumors, and to reproduce the molecular interplay between sarcoma cells and stromal surrounding cells (Troy et al., 2021; Joyce et al., 2021; Fan et al., 2022; Terzopoulou et al., 2022; Filippi et al., 2020; Nikolova and Chavali, 2019; Jenkins and Little, 2019; Weißenbruch et al., 2021).

Natural biological materials for 3D sarcoma cells culture include the use of Matrigel^®^ or sponges obtained by mixil natural monomers (Moizhess and Vasil’ev, 2013), acellular natural matrix (decellularized ECM) (Arca et al., 2011) and chitosan (Shalumon et al., 2011). In this regard, the purpose of using natural biological components aims to improve the features of the *in vitro* microenvironment obtaining an increase in tumor cells attachment and differentiation. Collagen-based gel matrix has been widely used with optimized protocols for providing physiologically relevant tissue stiffness and ECM composition. It includes laminin, collagen IV, heparan sulfate proteoglycans, entactin/nidogen, and a number of growth factors. Hamdi et al. (2015) and colleagues reported the characterization of chondrosarcoma cells within 3D collagen scaffolds and their response to radiation. The results showed that the 3D matrix allowed to recreate a microenvironment similar to the *in vivo* one, underlying the discrepancy which subsists between standard flat culture and 3D culture models on radiotherapy reactions. Recent works have stressed the validity of collagen-based scaffold sponges in reproducing tissue morphology and drug responsivity compared to 2D standard cultures. De Vita and colleagues have demonstrated that 3D collagen-based scaffold could provide useful platforms for diagnostic purpose and for studying the biological behavior of liposarcoma (Liverani et al., 2017), myxofibrosarcoma (De Vita et al., 2017a; Miserocchi et al., 2018) and undifferentiated pleomorphic sarcoma (UPS) (De Vita et al., 2017b). Furthermore, they have deepened the mechanism of action of chemotherapeutics currently used or under clinical investigation in rhabdomyosarcomas (De Vita et al., 2021a), in UPS and L-sarcoma (De Vita et al., 2021b), and in Giant Cell Tumor of Bone and Desmoplastic Fibroma (De Vita et al., 2022b). Acellular natural matrices obtained *via* enzymatic digestion of the cells from a tissue have been proved to be useful in retaining some of the structure and biomechanical functions of the origin tissue. Acellular cancellous bone grafts have been obtained by Arca and colleagues who proved that they have similar behavior to the osteosarcoma cell line MG63 (Arca et al., 2011). Similar results were obtained through decellularization of porcine jejunal segment derived scaffold which exhibited a tumor-like tissue microenvironment with the seeding of malignant peripheral nerve sheath tumors (MPNSTs) (Moll et al., 2013). Furthermore, the use of chitosan, a natural polysaccharide derived from chitin with biocompatibility, biodegradability, and osteoinductive properties, thus particularly exploited in bone tissue engineering applications, has been established for 3D porous scaffold research applications. Tan et al. (2014) and colleagues confirmed its non-toxicity on osteoblasts and chondrosarcoma cells.

Bioresorbable synthetic polymeric biomaterials have been used to design 3D scaffolds, mainly through extrusion printing and laser-assisted bioprinting techniques, as promising substitutes for the natural ECM proteins thanks to their high versatility, reproducibility, good processability together with their biophysical and biochemical characteristics. These features have attracted the interest on their use, especially due to their suitability for standardized manufacturing. Several works have demonstrated their biocompatibility, associated to good mechanical properties that can be advantageously tailored depending on the specific application. An example is the use of poly (D,L-lactic acid) based-scaffold which was able to sustain the growth of human osteosarcoma-derived osteoblast cell line (MG63) (Stoppato et al., 2013). Polycaprolactone (PCL) scaffolds have been tested with good results in terms of cell viability and for drug screening in Ewing sarcoma within a flow perfusion bioreactor (Santoro et al., 2015) and for post-surgical infections in sarcoma osteogenic-2 (Saos-2) cells (Comini et al., 2021). Recently, a composite hydrogel-electrospun nanofiber scaffold based on Poly (Ethylene Oxide)/Poly (Butylene Terephthalate) (PEOT/PBT) has been proposed as a promising implantable device for STS local therapy and tissue regeneration (Liguori et al., 2022). Polyetheretherketone bone scaffolds have been synthesized by 3D printing technologies with bioactive hydroxyapatite coating and anti-cancer drugs for osteosarcoma treatment (Zhu et al., 2021). Hierarchically Porous Osteoinductive Poly (hydroxyethyl methacrylate-*co*-methyl methacrylate) have been loaded with doxorubicin for local treatment in Osteosarcoma and bone defect repair (Sreeja et al., 2021).

## 4 Nanomedicine in the sarcoma patients’ clinical management and under clinical investigations

Taking in consideration the nanomedicines portfolio for sarcoma patient’s management, little steps forward have been carried out in the last decades. In this regard, among almost twenty nanomedicines currently approved for cancer treatment by FDA and listed in NIH (https://www.cancer.gov/nano/cancer-nanotechnology/current-treatments), only four are approved for sarcoma patients. Three of these are approved for the treatment of AIDS-related Kaposi’s sarcoma as Doxil (Caelyx)^®^ and Kaposi’s sarcoma as DaunoXome^®^ and Lipo-Dox^®^. Liposomal mifamurtide (MEPACT) is approved for the treatment of Osteosarcoma. No other sarcoma histotypes are currently included in already approved drug delivery systems. Starting from this, some clinical trials have been proposed to assess the role of nanotherapeutics in sarcoma patients.

### 4.1 Completed clinical trials

A phase II focusing on the use of pegylated liposomes loading doxorubicin in advanced STS was reported by Chidiac (Chidiac et al., 2000). Response and toxicity were evaluated in 15 patients treated with a schedule of 50 mg/m2 every 4 weeks. The most common histotypes were leiomyosarcoma (7/15) and UPS (2/15). The results showed that no patients experienced objective response. No lethal toxicity occurred and 20% of patients experienced grade 3–4 leukopenia or neutropenia. Thus, no significant therapeutic activity with this dose and schedule was observed, and the study warranted further investigations using Doxil^®^ at different doses and schedules for advanced STS. In 2010 a phase I study assessed the maximum tolerated dose (MTD) and safety of non-pegylated liposomes Myocet and ifosfamide in patients with metastatic STS (Stroppa et al., 2010). Ten patients were enrolled in the study and eight were treated. The results showed that the combination of intravenous Myocet^®^ 40 mg/m2 and ifosfamide 3,000 mg/m2 was well tolerated and phase II study was recommended. A more robust clinical trial comparing the activity of Caelyx^®^/Doxil^®^ pegylated liposomal doxorubicin (50 mg/m2) *versus* standard treatment doxorubicin (75 mg/m2) was carried out (Judson et al., 2001). A total of 94 patients affected by metastatic STS were enrolled. The most common histotype was leiomyosarcoma with 33% proportion among the case series (Caelyx^®^: 18; doxorubicin: 13). The results showed equivalent activity between Caelyx^®^ and doxorubicin (Caelyx^®^: complete response 1 (uterine), partial response 4 (response rate 10%); doxorubicin: complete response 1, partial response 3 (response rate of 9%), stable disease (SD) in 16 and 18 patients, respectively). The authors concluded that Caelyx^®^ should be considered for further investigation in combination with other chemotherapeutic agents including ifosfamide. Considering the non decisive studies based on the use of DDS in sarcoma patients described above, a story of success is represented by AIDS-related Kaposi’s sarcoma and DaunoXome^®^. In this regard a randomized phase III trial study comparing the safety and efficacy of liposomal daunorubicin DaunoXome^®^ with a reference regimen of doxorubicin, bleomycin, and vincristine (ABV) lead to the approval of this DDS by FDA in 1996 (Gill et al., 1996). A total of 232 patients were enrolled and 227 were treated (116 with DaunoXome and 111 with ABV). The results showed a comparable efficacy of DaunoXome^®^ and ABV with a median survival time of 369 days for DaunoXome^®^ and 342 days for ABV and a manageable toxicity profile. Moreover, the median time to treatment failure was 115 days for DaunoXome^®^ and 99 days for ABV. Thus, DaunoXome^®^ was approved as an effective and safe therapy for advanced AIDS-related Kaposi’s sarcoma. In 2020 albumin-bound paclitaxel nanoparticle Abraxane^®^ and gemcitabine combination has been evaluated for STS treatment in a retrospective observational study (Tian et al., 2020). The study enrolled 17 patients affected by STS previously treated with anthracyclines-based regimen. The results showed a complete response in one angiosarcoma patient, six patients experienced partial response, five patients achieved stable disease and five progressive diseases. The authors concluded that nab-paclitaxel/gemcitabine combination chemotherapy was comparatively effective in the treatment of STS and that is worthy of further study.

From the point of view of bone sarcomas, osteosarcoma represents the most common histotype. In 2005 a randomized, prospective trial evaluated if the combination of ifosfamide and/or muramyl tripeptide (MTP) delivered by liposomes and cisplatin, doxorubicin, and high-dose methotrexate (HDMTX) could improved the probability for event-free survival (EFS) in patients with osteosarcoma (Meyers et al., 2005). A total of 677 patients with primary osteosarcoma were included in the study. The results showed that the addition of ifosfamide to standard chemotherapy did not produce any advantage in terms of EFS. On the contrary, the addition of MTP could improve EFS, however further research is needed in order to deepen the interactions between ifosfamide and MTP.

Finally, a the role of a first-in-class radioenhancer hafnium oxide nanoparticle has been evaluated in a phase I (Bonvalot et al., 2017) and in a multicentre, phase 2-3, randomised, controlled trial (Bonvalot et al., 2019) for locally advanced STS. This phase 2-3 study evaluated the safety and efficacy of the hafnium oxide (HfO_2_) nanoparticle NBTXR3 activated through radiotherapy *versus* radiotherapy alone as a pre-operative treatment in patients with locally advanced STS. One hundred patients were enrolled and randomly assigned and 179 were treated (89 with NBTXR3 plus radiotherapy and 90 with the radiotherapy alone). A complete response was observed in 14 (16%) of 87 patients in the NBTXR3 group and in 7 (8%) of 89 in the radiotherapy alone group (*p* = 0.044). Thus, the trial validated this new class of radioenhancer for the clinical application in STS and opened its potential application to other cancers.

A summary of the closed clinical trials assessing the role of DDS in sarcoma described above is reported in Table 1.

**TABLE 1:** Closed clinical trials assessing the role of DDS in sarcoma.

Study	Formulation	Chemotherapy	Dosage	Lesions	References
Albumin-bound paclitaxel and gemcitabine combination therapy in soft tissue sarcoma	Albumine-based nanoparticles	Paclitaxel and gemcitabine combination	300 mg/m2 nab-paclitaxel *via* IV bolus on D1, 1,250 mg/m2 gemcitabine *via* IV bolus on D1 and D8	STS	Tian et al. (2020)
Phase II trial of liposomal doxorubicin (Doxil) in advanced soft tissue sarcomas	Pegylated liposomes	Doxorubicin	50 mg/m2 every 4 weeks	STS	Chidiac et al. (2000)
Phase I study of non-pegylated liposomal doxorubicin in combination with ifosfamide in adult patients with metastatic soft tissue sarcomas	Non-pegylated liposomes	Doxorubicin	Up to five cycles of IV ifosfamide 3,000 mg/m2 on D1- 3 in combination with escalating doses of IV Myocet on D1 every 3 weeks until DLT in at least one patient. Starting dose of Myocet: 40 mg/m2 to be escalated through 10 mg/m2 increase up to 80 mg/m2	STS	Stroppa et al. (2010)
Randomised phase II trial of pegylated liposomal doxorubicin (DOXIL/CAELYX) *versus* doxorubicin in the treatment of advanced or metastatic soft tissue sarcoma: a study by the EORTC Soft Tissue and Bone Sarcoma Group	Pegylated liposomes	Doxorubicin	CAELYX (50 mg/m (2) by a 1 h IV infusion every 4 weeks vs. doxorubicin (75 mg/m2 by an IV bolus every 3 weeks	STS	Judson et al. (2001)
Randomized phase III trial of liposomal daunorubicin *versus* doxorubicin, bleomycin, and vincristine in AIDS-related Kaposi’s sarcoma	Liposomes	Daunorubicin	DaunoXome 40 mg/m2 or a combination regimen of doxorubicin 10 mg/m2, bleomycin 15 U, and vincristine 1 mg, administered IV every 2 weeks	Kaposi’s sarcoma	Gill et al. (1996)
Osteosarcoma: a randomized, prospective trial of the addition of ifosfamide and/or muramyl tripeptide to cisplatin, doxorubicin, and high-dose methotrexate					
Osteosarcoma: a randomized, prospective trial of the addition of ifosfamide and/or muramyl tripeptide to cisplatin, doxorubicin, and high-dose methotrexate	Liposomes	Muramyl tripeptide phosphatidylethanolamine; L-MTP-PE	Cisplatin 120 mg/m2 combined with doxorubicin 75 mg/m2 administered twice during induction at weeks 0 and 5 and twice during maintenance at weeks 12 and 17. Additional two courses of doxorubicin without cisplatin administered at weeks 22 and 27. HDMTX 12 g/m2 followed by leucovorin 10 mg beginning 24 h from initiation of methotrexate infusion	Osteosarcoma	Meyers et al. (2005)
NBTXR3, a first-in-class radioenhancer hafnium oxide nanoparticle, plus radiotherapy *versus* radiotherapy alone in patients with locally advanced soft-tissue sarcoma (Act.In.Sarc): a multicentre, phase 2–3, randomised, controlled trial	First-in-class radioenhancer hafnium oxide nanoparticle	NBTXR3	NBTXR3 (volume corresponding to 10% of baseline tumour volume at a fixed concentration of 53·3 g/L) as a single intratumoural administration before preoperative external-beam radiotherapy (50 Gy in 25 fractions) or radiotherapy alone, followed by surgery	Locally advanced soft-tissue sarcoma	Bonvalot et al. (2017)
					Bonvalot et al. (2019)

### 4.2 Ongoing clinical trials

Few studies are currently ongoing assessing the role of DDS in sarcoma. A phase I dose-escalation study of nab-Rapamycin and pazopanib followed by a phase II study is currently carried on (NCT03660930) in advanced and metastatic STS. The estimated enrollment is 57 participants and the end date is scheduled for february 2025. A phase I trial is assessing the side effects and best dose of nab-paclitaxel and bevacizumab in patients with unresectable stage IV melanoma or gynecological cancers including uterine sarcoma (NCT02020707). A total of 73 patients are expected to be enrolled and the estimated end date is June 2025. Due to the increasing evidence that the combination therapy with nab-paclitaxel and programmed cell death protein 1 (PD-1) inhibitor is promising in treating different tumors, a phase II study assessing the combination activity of nab-paclitaxcel Plus camrelizumab for advanced STS in currently ongoing (NCT05189483). The primary end point will be progression-free survival at 4 months and secondary objectives include objective response rate and safety. Fourteen patients will be enrolled and the estimated study completion date is July 2023 (NCT05189483). Moreover, a recent phase I study is focusing on the use of superparamagnetic iron oxide nanoparticles and spinning magnetic field (SPIONs/SMF) for osteosarcoma (NCT04316091). In particular, the study aims to evaluate the safety, efficacy, and tolerability of SPIONs/SMF in combination with neoadjuvant chemotherapy in osteosarcoma patients. The study is not yet recruiting and 60 patients will be included. The estimated end date is august 2023.

A summary of the ongoing clinical trials assessing the role of DDS in sarcoma described above is reported in Table 2.

**TABLE 2:** Ongoing clinical trials assessing the role of DDS in sarcoma.

Ongoing clinical trial	Formulation	Chemotherapy	Dosage	Lesions	Clinical trial identifier
Nanoparticle Albumin-Bound Rapamycin and Pazopanib Hydrochloride in Treating Patients With Advanced Nonadipocytic Soft Tissue Sarcomas	Nanoparticle Albumin-Bound	Rapamycin	Nab-pamycin (IV) on D1 and D8 and pazopanib hydrochloride orally (PO) daily on days 1–21. Cycles repeat every 21 days	—Advanced STS	NCT03660930
				—Locally Advanced STS	
				—Metastatic STS	
Nab-Paclitaxel and Bevacizumab in Treating Patients With Unresectable Stage IV Melanoma or Gynecological Cancers	Albumine-based nanoparticles	Paclitaxel	Nab-paclitaxel/bevacizumab-complex intravenously (IV) on D1, D8, and D15. Cycles repeat every 28 days. Patient may receive paclitaxel if supply of nab-paclitaxel is exhausted	—Cervical Adenosarcoma	NCT02020707
				—Cervical Carcinosarcoma	
				—Fallopian Tube	
				—Carcinosarcoma	
				—Ovarian Carcinosarcoma	
				—Primary Peritoneal	
				—Carcinosarcoma	
				—Uterine Corpus	
				—Carcinosarcoma	
Albumin-bound Paclitaxel Plus Camrelizumab for Advanced Soft Tissue Sarcoma	Albumine-based nanoparticles	Paclitaxel	300 mg/m2 of nab-paclitaxel and 200 mg of PD-1 inhibitor *via* a 30-min IV infusion on D1. Treatment was repeated every 3 weeks	STS	NCT05189483
Phase I Clinical Trial of Neoadjuvant Chemotherapy With/Without SPIONs/SMF for Patients With Osteosarcoma	Superparamagnetic Iron Oxide Nanoparticles	Conventional neoadjuvant chemotherapy	Intratumoral injection of SPIONs every other day for 3 times, followed by SMF every 2 days, and up to completion of 30 days, and conventional neoadjuvant chemotherapy from D1	Osteosarcoma	NCT04316091

### 4.3 Challenges and possible drawbacks of using nanomedicine to treat sarcoma

Although nanomedicines represent a promising strategy to increase the therapeutic portfolio for sarcoma treatment, especially in the era of personalized medicine, they are not free from limitations in terms of toxicity and sustainability. In this regard, nanomedicine is becoming an attractive field of research for the pharmaceutical industry due to its higher efficacy compared to conventional chemotherapy and to the lower amount of compounds needed with an impact on drug manufacturing (Rasool et al., 2022). Otherwise, together with an enhanced drug bioavailability, targeting and uptake of some compounds used for DDS synthesis including among all aluminum oxide gold, copper oxide, silver, zinc oxide, iron oxide, titanium oxide, graphene oxide, carbon, fullerene, silica, are associated with toxicities (Karmakar et al., 2014). Moreover, even if biodegradable or polymeric materials are non-toxic, non-immunologic and non-inflammatory, some research has proposed that the NPs surface decoration could lead to toxicity through the activation of macrophages (Grabowski et al., 2015).

Furthermore there are evidences that nano-sized drug delivery systems can suffer from selective organ toxicity due to their tissue accumulation (Nel et al., 2006) which is dependent on the NPs administered concentration. Another theme which needs to be taken into account is represented by the specific anatomy districts interested by sarcoma lesions that are challenging to be targeted by DDS due to their reduced blood perfusion (i.e., adipose tissue, synovial tissue, connective tissue, smooth muscle tissue, skeletal tissue) limiting their application in comparison to other solid and hematological malignancies. In addition to these drawbacks one major limitation is represented by the high production cost and technology needed for DDS development and the possible smaller profit of pharmaceutical industry in comparison to big killer tumors.

## 5 Future outlooks

As closing remarks, it is a fact that the role of nanotechnology in cancer application and in particular in the field of sarcoma is growing. On the other hand, an increased effort in preclinical and clinical research on this topic is needed in order to rapidly translate the observations into clinical practice (Kemp and Kwon, 2021). Several studies have demonstrated the benefits of nanotechnology in cancer treatment, imaging, and diagnostics but the evaluation of cost analysis including manufacturing together with unclear regulatory guidelines have posed many questions and in part have limited the growth of this approach. Future perspectives are represented by the improvement of early detection methods for sarcoma lesions which could have potentially a huge impact on patient’s outcome. At the same time, the broad heterogeneity of sarcoma lesions requires dedicated DDS design for each specific type of tumor and - importantly - specific models to study the bio-nano interaction at all levels, from the cell to the tissue and to the trafficking properties. Moreover, the delivery of conventional cancer therapies and radiotherapies, including specifically targeted technologies, together with the development of novel nanomaterials for further enhanced cancer immune therapies are some of the future fields of application in which nanotechnology could play a pivotal role. In conclusion, the emerging role of sarcoma nanotechnology could promise to enhance the management of these complex diseases and to prevail on their dynamic nature (Weißenbruch et al., 2021).
